# Frequent premature atrial contractions as a signalling marker of atrial cardiomyopathy, incident atrial fibrillation, and stroke

**DOI:** 10.1093/cvr/cvac054

**Published:** 2022-04-07

**Authors:** José Maria Farinha, Dhiraj Gupta, Gregory Y H Lip

**Affiliations:** Liverpool Centre for Cardiovascular Science, University of Liverpool and Liverpool Heart & Chest Hospital, Liverpool, UK; Liverpool Centre for Cardiovascular Science, University of Liverpool and Liverpool Heart & Chest Hospital, Liverpool, UK; Liverpool Centre for Cardiovascular Science, University of Liverpool and Liverpool Heart & Chest Hospital, Liverpool, UK; Department of Clinical Medicine, Aalborg University, Aalborg, Denmark

**Keywords:** Anticoagulation, Atrial cardiomyopathy, Atrial myopathy, Premature atrial contractions, Supraventricular ectopic beats, Stroke

## Abstract

Premature atrial contractions are a common cardiac phenomenon. Although previously considered a benign electrocardiographic finding, they have now been associated with a higher risk of incident atrial fibrillation (AF) and other adverse outcomes such as stroke and all-cause mortality. Since premature atrial contractions can be associated with these adverse clinical outcomes independently of AF occurrence, different explanations have being proposed. The concept of atrial cardiomyopathy, where AF would be an epiphenomenon outside the causal pathway between premature atrial contractions and stroke has received traction recently. This concept suggests that structural, functional, and biochemical changes in the atria lead to arrhythmia occurrence and thromboembolic events. Some consensus about diagnosis and treatment of this condition have been published, but this is based on scarce evidence, highlighting the need for a clear definition of excessive premature atrial contractions and for prospective studies regarding antiarrhythmic therapies, anticoagulation or molecular targets in this group of patients.

## Graphical Abstract

**Graphical Abstract:**
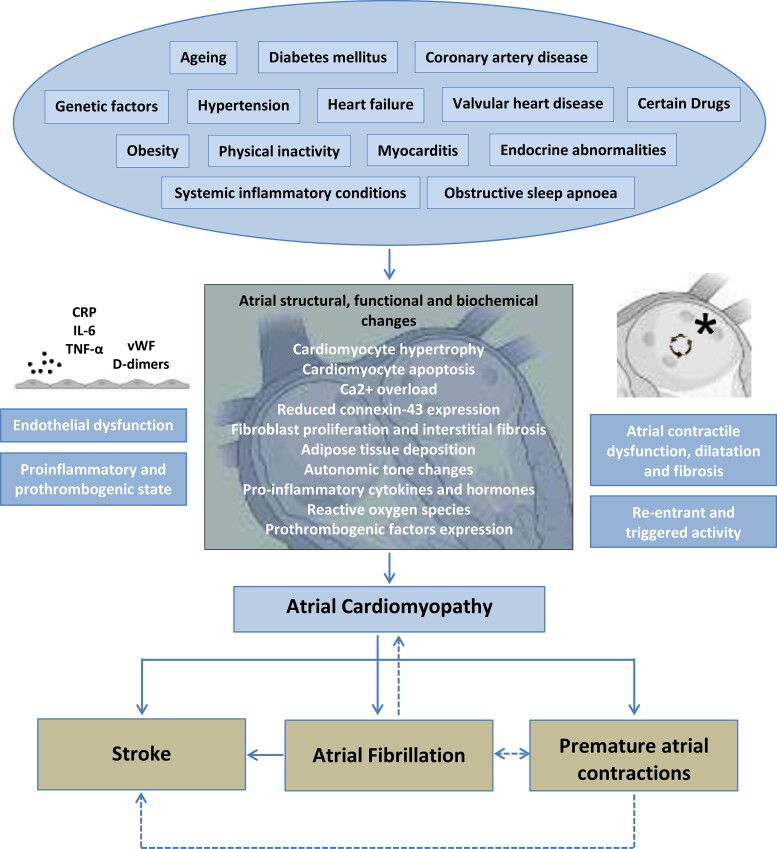
The concept of atrial cardiomyopathy and its association with atrial ectopy, atrial fibrillation, and stroke. Atrial cardiomyocytes and non-cardiomyocyte elements react briskly and extensively to pathological stimuli and are susceptible to a range of genetic influences, leading to structural, functional, and biochemical changes that will have a substantial impact on cardiac performance, arrhythmia occurrence, and stroke risk. Created with figures from BioRender.com. CRP, C-reactive protein; IL-6, interleukin 6; TNF-α, tumour necrosis factor alpha; vWF, von Willebrand factor.

## 1. Introduction

Premature atrial contractions (PACs) are a common cardiac phenomenon across all age groups, increasing with advancing age.^1,2^ PACs were previously considered a benign electrocardiographic finding, but this notion has been changing since PACs have been associated with a higher risk of incident atrial fibrillation (AF) and other adverse outcomes, especially stroke.^1–3^

Three major explanations have been proposed for this association. One suggests that the presence of frequent PACs identifies patients likely to develop AF, which leads to an increased risk of stroke and death.^1,3–5^ Another suggests that increased atrial ectopy is frequently associated with traditional risk factors for cardiovascular disease.^1,3,5^ One more explanation suggests that frequent PACs themselves may be a marker for atrial cardiomyopathy, which may be directly related to the development of AF and to an increased stroke risk.^1,3–5^ Strengthening this last hypothesis has been some recent genetic studies showing an association between mutations in certain genes and the development of AF.^4,5^

This positive relation between PACs and subsequent risk of incident AF or stroke highlights the importance of finding a clinically relevant threshold for PACs frequency that could trigger more stringent AF screening or even the necessity for anticoagulation and/or radiofrequency ablation in the absence of actual documented AF.^1–4^

The objective of this review is to provide an overview of the evidence for frequent PACs as a signalling marker of atrial cardiomyopathy, incident AF, and stroke.

## 2. Search strategy

We searched the PubMed database for relevant reviews and original research studies (for full search strategy, see Supplemental material online).

## 3. Epidemiological insights

PACs are highly prevalent and become more frequent with advancing age. A cross-sectional analysis of a random sample of adults from the general population aged ≥50 years who underwent 24 h Holter monitoring found that 99% of participants had at least one PAC per day, with a median number of PACs per hour increasing from 0.8 (0.4–1.8) in those aged 50–55 years to 2.6 (1.2–6.5) in those aged ≥70 years. Furthermore, individuals with established cardiovascular disease had a two-fold greater number of PACs per hour.^6^

The published evidence has generally suggested a positive relation between frequency of baseline PACs and risk of incident AF, stroke, and all-cause mortality (*Table 1*; see Supplementary material online, *Tables S1–S3*).^1–3^

**Table 1:** Recent systematic reviews and meta-analysis on frequent PACs and their association with adverse outcomes

Author, year	Studies included in the meta-analysis	Studies designs	Total number of patients	Mean age range, in years	Male gender range, in %	Baseline recording device	Follow-up range, in years	Definition of PAC count as the predictor	Studied outcomes	Effect measure (95% CI) of the association between PAC count and studied outcome^b^
**Meng *et al.*, 2020^1^**	^7,10–13^	Three prospective cohort studies^7,10,11^ Two retrospective cohort studies^12,13^	7545	61.8 ± 18.6^13^ to 71.3^10^	43.7^11^ to 92.9^12^	48 h Holter^7^ 24 h Holter^10–13^	6.1 ± 1.3^11^ to 10.0 ± 1.0^13^	ESVEA was defined as > 30 PACs per hour and/or runs of ≥20 PACs or > 218 PACs per 24 h	AF^7,10–13^	Adjusted HR (95% CI): 2.19 (1.70–2.82)
									Stroke^7,11^	Adjusted HR (95% CI): 2.23 (1.24–4.02)
									All-cause mortality^7,10,11,13^	Adjusted HR (95% CI): 1.61 (1.25–2.07)
**Himmelreich *et al.*, 2018^2^**	^7,10–25^	Twelve prospective cohort studies^7,10,11,14,16–18,20,21,23–25^ Five retrospective cohort studies^12,13,15,19,22^	17 983	54.2 ± 15.5^14^ to 75.8 ± 5.3^23^	43.7^11^ to 100.0^23^	48 h Holter^7,15,16^ 24 h Holter^10–14,17–22,24,25^ Polysomnography continuous ECG^23^	0.9 ± 0.3^18^ to 13.0 (7.3–18.1)^24^	Any dichotomized baseline PAC count	AF^7,10–23^	Unadjusted HR (95% CI): 2.96 (2.33–3.76)
									Stroke^7,10,11^	Unadjusted HR (95% CI): 2.54 (1.68–3.83)
									All-cause mortality^7,10,11,13,15,16^	Unadjusted HR (95% CI): 2.14 (1.94–2.37)
								Any categorization of baseline PAC count	AF^10,21,23–25^	Unadjusted HR (95% CI): 3.93 (2.53–6.09)
								Continuous PAC count on any scale	AF^7,10,16,22–24^	Unadjusted HR (95% CI): 1.57 (1.42–1.74)
									All-cause mortality^7,10,16,24^	Unadjusted HR (95% CI): 1.39 (1.30 – 1.48)
**Huang *et al.*, 2017^3^**	^8,11,13,26–33^	9 prospective cohort studies^8,11,26–31,33^ 2 retrospective cohort studies^13,32^	129 514	52.5 ± 13.7^26^ to 68.0^8c^	32.4^28^ to 100.0^8^	48 h Holter^27^ 24 h Holter^8,11,13,32^ Routine screening 12-lead ECG^26,30,31^ 15 s ECG^28^ 2 min ECG^29,33^	6.1 ± 1.3^11d^ to median of 14.4^27^	Dichotomized as frequent PACs vs. no frequent PACs^a^	Cardiovascular death^13,26,28,31^	Unadjusted RR (95% CI): 2.89 (2.20–3.79)^13,26,28,31^ Adjusted RR (95% CI): 1.38 (1.24–1.54)^26,28,31^
									Death from coronary artery disease^13,26,31–33^	Unadjusted RR (95% CI): 2.74 (1.64–4.58)^13,26,31–33^ Adjusted RR (95% CI): 1.74 (1.27–2.37)^31,33^
									Stroke^8,11,13,26–30^	Unadjusted RR (95% CI): 2.20 (1.79–2.70)^8,11,13,26–30^ Adjusted RR (95% CI): 1.41 (1.25–1.60)^8,27–30^
									All-cause mortality^8,11,13,26,28,31^	Unadjusted RR (95% CI): 2.17 (1.80–2.63)^8,11,13,26,28,31^ Adjusted RR (95% CI): 1.26 (1.13–1.41)^8,13,26,28,31^

Any presence of PAC was defined as exposure in all studies using ECG, whereas the definitions of frequent PACs in studies using 24 or 48 h Holter monitoring were so diverse as ≥30% of the recording time, ≥218 PACs/24 h, >100 PACs/24 h, ≥30 PACs/h, and >76 PACs/24 h.

Where adjusted results are presented, the meta-analysis was also performed including the effect measures obtained after adjusting for confounding variables in each individual study.

Not including the study from Algra *et al.*,^32^ which dichotomized age as <60 and ≥60 years (50.3% of patients in the group ≥60 years).

Excluding the study from Algra *et al.*,^32^ which followed the patients for 2 years period but did not state the mean/median follow-up (follow-up was complete in 99.5% of the patients and 10.7% of the patients died).

AF, atrial fibrillation; PAC(s), premature atrial contraction(s).

Until now, a threshold value of PACs frequency in predicting AF or other adverse outcomes has not been agreed upon.^1,3,4^ The term ‘excessive supraventricular ectopic activity’ (ESVEA) has been used to describe different manifestations of intense atrial ectopy, although without a consensus definition.^1^

### 3.1 Different recording methods and different definitions of frequent PACs

In the majority of studies concerning PACs and cardiovascular outcomes, 24 or 48 h Holter monitoring is the most frequent method for PACs screening. Although routine 12-lead ECG, 15 s ECG, 2 min ECG, exercise test continuous ECG, and polysomnography continuous ECG have also been used, Holter monitoring has been considered the most reliable method for PACs burden characterization and for cardiovascular outcomes prediction.^1–3^

In a recent systematic review and meta-analysis performed by Meng *et al.*^1^, ESVEA was defined with continuous monitoring as >30 PACs per hour and/or runs of ≥20 PACs or >218 PACs per 24 h, in accordance with previous studies.^7–10^

Another systematic review and meta-analysis performed by Himmelreich *et al.*^2^ did not find a consensual definition for excessive PACs. Considering continuous monitoring, some of the included studies reported on similar cut-offs for dichotomization, whether by coincidence (e.g. 100 PACs/24 h) or by design (e.g. cut-off = ESVEA as defined by Binici *et al.*^7^, which is ≥30 PACs per hour or any episode of runs of ≥20 PACs). Other studies based their cut-off on a percentile of PAC count (e.g. cut-off = lower bound of upper quartile) or on the derived optimum for outcome prediction within the cohort, whereas others provided no justification for the chosen cut-off value.^2^

A systematic review and meta-analysis by Huang *et al.*^3^ also found different definitions of frequent PACs. Any presence of PAC was defined as exposure in studies using ECG, whereas the definitions of frequent PACs in studies using Holter monitoring were so diverse as ≥30% of the recording time, ≥218 PACs/24 h, >100 PACs/24 h, ≥30 PACs/h, and >76 PACs/24 h.

Despite the heterogeneity of the included studies, with follow-up times ranging between a mean of 1 year and 14 years, these three systematic reviews and meta-analyses concerning PACs and cardiovascular outcomes covered a similar follow-up range and reached comparable conclusions (*Table 1*).^1–3^

### 3.2 Frequent PACs and incident AF

Meng *et al.*^1^ investigated the potential association between ESVEA and the risk of AF. The pooled analysis of five different studies using Holter monitoring^7,10–13^ showed that ESVEA was associated with a two-fold adjusted increased risk of incident AF (*Table 1*; see Supplementary material online, *Table S1*).^1^

Regarding the association between PACs and incident AF, Himmelreich *et al.*^2^ performed three different analyses. The meta-analysis of all 15 studies that reported on any dichotomized baseline PAC count and its association with AF^7,10–23^ showed an almost three-fold unadjusted increased risk of incident AF for those patients with a higher PAC count,^2^ the meta-analysis of all five studies that applied any categorization of baseline PAC count as an ordinal variable to predict AF^10,21,23–25^ resulted in an almost four-fold unadjusted increased risk,^2^ and the meta-analysis of all six studies that considered continuous PAC count on any scale and its association with AF^7,10,16,22–24^ resulted in an almost two-fold unadjusted increased risk (*Table 1*; see Supplementary material online, *Table S1*).^2^

Thus, patients with a higher PAC count were shown to have a two- to four-fold increased risk of incident AF, depending on the PAC count measure and adjustments for confounding variables.^1,2^

### 3.3 Frequent PACs and stroke risk

In the systematic review and meta-analysis by Meng *et al.*,^1^ two studies assessed the association between ESVEA and the risk of stroke.^7,11^ Overall, ESVEA was associated with a two-fold adjusted increased risk of stroke (*Table 1*; see Supplementary material online, *Table S2*).^1^

The meta-analysis by Himmelreich *et al.*^2^ included three studies^7,10,11^ that reported on dichotomized PAC count for the outcome first stroke, resulting in an almost three-fold unadjusted increased risk for those patients with a higher PAC count (*Table 1*; see Supplementary material online, *Table S2*).^2^

Regarding the risk of stroke, Huang *et al.*^3^ reported a similar two-fold increased risk for those patients with frequent PACs when considering eight studies that reported unadjusted estimates^8,11,13,26–30^ but only a 41% increased risk when considering only those five studies adjusting for potential confounding factors (*Table 1*; see Supplementary material online, *Table S2*).^8,27–30^

Thus, patients with a higher frequency of PACs were shown to have a two-fold to almost three-fold increased risk of first stroke after dichotomization of PAC count.^1–3^ However, this effect may be diminished after considering other cardiovascular risk factors.^1,3^

### 3.4 Frequent PACs and all-cause mortality

In the systematic review and meta-analysis by Meng *et al.*,^1^ four studies assessed the association between ESVEA and all-cause mortality.^7,10,11,13^ The pooled analysis showed that ESVEA increased the adjusted risk of all-cause mortality by 61% (*Table 1*; see Supplementary material online, *Table S3*).^1^

On the association between PACs and all-cause mortality, Himmelreich *et al.*^2^ performed two different analyses. The meta-analysis of all six studies reporting on the relationship between dichotomized PAC count and all-cause mortality^7,10,11,13,15,16^ found a two-fold unadjusted increased risk for those patients with a higher PAC count,^2^ and the meta-analysis of all four studies on continuous PAC count and its association with all-cause mortality^7,10,16,24^ resulted in a 39% unadjusted increased risk (*Table 1*; see Supplementary material online, *Table S3*).^2^

Huang *et al.*^3^ also reported a similar finding concerning all-cause death. Patients with frequent PACs presented a two-fold increased risk for all-cause mortality when considering six studies presenting unadjusted estimates,^8,11,13,26,28,31^ but only a 26% increased risk when considering only those five studies adjusting for confounders (*Table 1*; see Supplementary material online, *Table S3*).^8,13,26,28,31^

Thus, patients with a higher PAC count were shown to have between 39% and two-fold increased risk of all-cause mortality, depending on the PAC count measure.^2^ However, this mortality risk may be reduced to just 26% after adjusting for individual risk factors.^1,3^

### 3.5 Frequent PACs and other cardiovascular outcomes

The meta-analysis by Huang *et al.*^3^ reported the relationship between PACs frequency and other cardiovascular outcomes besides stroke and all-cause mortality.

The pooled analysis of all four studies that reported the association between PACs and cardiovascular death^13,26,28,31^ revealed an almost three-fold unadjusted increased risk for those patients with a higher PAC count, but only three of these studies^26,28,31^ reported adjusted values, with an overall adjusted risk increased by only 38% (*Table 1*; see Supplementary material online, *Table S4*).^3^

The pooled analysis of all five studies that evaluated the association of PACs and death from coronary artery disease^13,26,31–33^ showed an almost three-fold unadjusted increased risk for those patients with a higher PAC count, but only two of these studies^31,33^ reported adjusted values, with an overall adjusted risk increased by 74% (*Table 1*; see Supplementary material online, *Table S5*).^3^

Thus, Huang *et al.*^3^ highlight that PACs can be a sign of non-cardiac diseases or indicators of subclinical cardiovascular diseases, which may diminish the independent association between PACs and adverse events.

## 4. Possible mechanisms behind the association of PACs with AF, stroke, and mortality

The evidence above included studies among patients without known AF either from cohorts of patients with recent cryptogenic stroke or from the general population. Although they identified an association between PACs and incident AF, they also showed that PACs can be associated with adverse clinical outcomes independently of AF.^1–3^

However, these studies could not establish causality nor definitively implicate atrial ectopy as the primary driver of increased risk of AF, stroke, or death. In addition, the identification of asymptomatic or paroxysmal AF is frequently difficult, making it impossible to exclude that some patients with frequent PACs also had previously non-diagnosed AF or that some patients who experienced stroke or death did not have intervening AF.^1–3^

This way, three different explanations for the association of PACs with AF, stroke, and mortality have been proposed. First, patients with a high burden of PACs are more likely to develop subclinical AF, which subsequently leads to an increased risk of stroke and death.^1,3,4^ Second, increased atrial ectopy has also been associated with traditional risk factors for cardiovascular disease including increasing age, lower HDL cholesterol levels, physical inactivity, existing cardiovascular disease, higher N-terminal Pro-B-type natriuretic peptide levels, obstructive sleep apnoea, and increased left ventricular filling pressures.^1,3,4,6,34^

Finally, is the concept of atrial cardiomyopathy, where AF would be an epiphenomenon outside the causal pathway between PACs and stroke.^1,3–5^ This concept derived from the finding that atrial cardiomyocytes and non-cardiomyocyte elements react briskly and extensively to pathological stimuli and are susceptible to a range of genetic influences, leading to structural, functional, and biochemical changes that will have a substantial impact on cardiac performance, arrhythmia occurrence, and stroke risk.^1,5,35–41^

### 4.1 The concept of atrial cardiomyopathy

The apparent inability of AF rhythm control strategies to eliminate stroke risk,^35,39,41–44^ and the lack of a strong temporal association between subclinical AF and stroke as assessed using implantable cardiac device monitoring have intensified the search for additional factors that could account for stroke beyond the rhythm disturbance itself,^35,38–40,45–47^ consequently leading to the concept of atrial cardiomyopathy.^35,38–40^

Blood stasis is no more the most considered factor for atrial derived thrombogenesis. Thrombus formation in atrial cardiomyopathies and AF is now known to also depend on endothelial injury and a hypercoagulability state, as suggested by Virchow’s triad.^36,48,49^

An international expert consensus document defined atrial cardiomyopathy as ‘any complex of structural, architectural, contractile or electrophysiological changes affecting the atria with the potential to produce clinically-relevant manifestations’.^5^

Many conditions or diseases are known to induce or contribute to an atrial cardiomyopathy, e.g. ageing, hypertension, obesity, diabetes mellitus, heart failure, valvular heart disease, coronary artery disease, myocarditis, systemic inflammatory conditions, certain drugs, endocrine abnormalities, genetic factors, obstructive sleep apnoea, and AF itself. Atrial cells, both cardiomyocytes and non-cardiomyocyte elements like fibroblasts, endothelial cells, and neurons, react to these pathological stimuli by cardiomyocyte hypertrophy or myocytolysis, changes in cardiomyocyte ion-channels and in connexin expression, atrial fibroblast proliferation and interstitial fibrosis, adipose tissue deposition, autonomic tone changes, release of pro-inflammatory cytokines and hormones, release of reactive oxygen species, and prothrombogenic factors expression (*Graphical Abstract*).^5,35,37–41,50^

The electrophysiological abnormalities seen in atrial cardiomyopathies include decreased atrial conduction velocity, reduced action potential duration, abbreviated refractoriness, increased fractionation, greater number of double potentials, and low-voltage areas.^5,40^

Cellular decoupling and myocyte isolation, tissue anisotropy, and conduction heterogeneities are considered the substrate for local re-entry.^5,35,40,41^ Abnormal Ca^2+^ handling is involved in the triggered activity and can promote beat-to-beat alternation in action potential duration that favours re-entry.^5,41,51^ Both triggered activity and re-entrant mechanisms are implicated in the genesis of atrial ectopy (*Graphical Abstract*).^52^

There is extensive evidence that AF initiation involves atrial ectopic triggers and a re-entry-prone substrate, with autonomic tone being a key regulator, and with abbreviated refractoriness being a potential contributor, particularly among individuals with gene variants that accelerate atrial repolarization.^5,38,41,50,51,53,54^ Thus, both ectopic activity and AF have a common background.^5,50–54^ Atrial enlargement and progressive fibrosis seen in atrial cardiomyopathies are also important determinants for the development and maintenance of AF (*Graphical Abstract*).^5,35,38,40,41,53,54^

**Figure 1:**
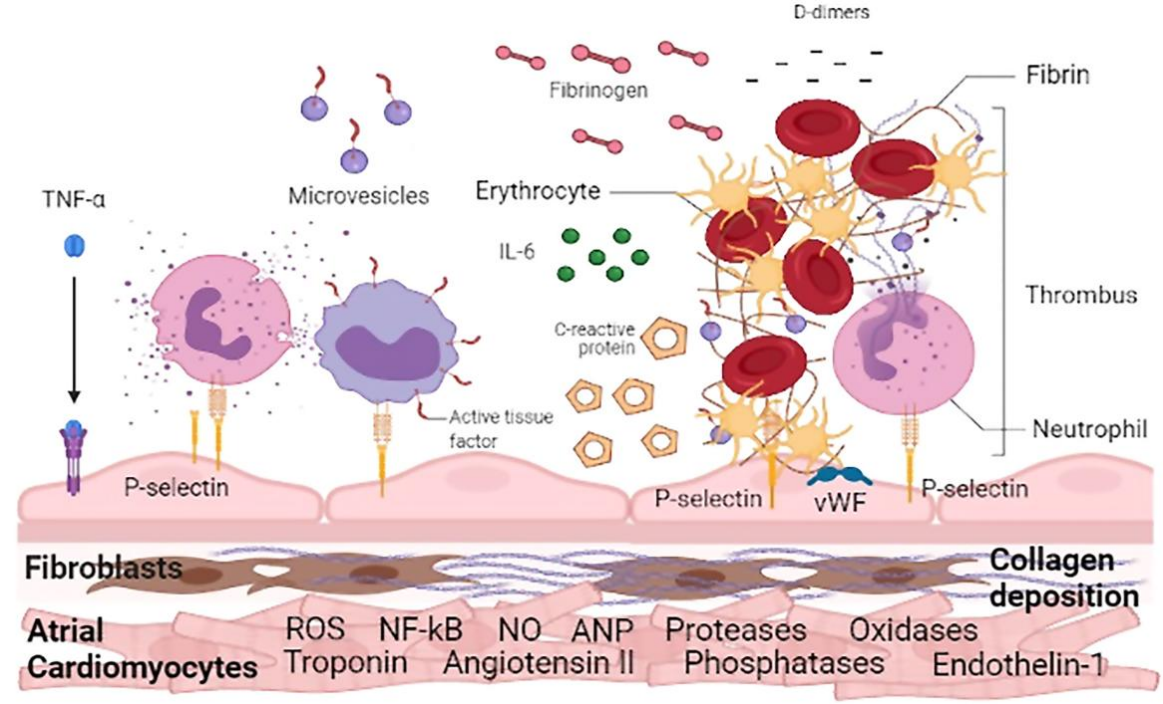
**Atrial cells reaction to pathological stimuli, leading to a hypercoagulable state.** Atrial cells react to pathological stimuli by cardiomyocyte changes, fibroblast proliferation, interstitial fibrosis, release of pro-inflammatory cytokines and hormones, release of reactive oxygen species, and expression of prothrombogenic factors. Created with BioRender.com. ANP, atrial natriuretic peptide; IL-6, interleukin 6; NF-kB, nuclear factor kappa-light-chain-enhancer of activated B cells; NO, nitric oxide; ROS, reactive oxygen species; TNF-α, tumour necrosis factor alpha; vWF, von Willebrand factor.

On the other hand, the response of atrial cells to pathological stimuli also leads to a hypercoagulable state. Atrial enlargement, fibrosis, and ion-current changes cause a contractile dysfunction that may be involved in thromboembolism. The presence of a pro-inflammatory state and the expression of prothrombogenic factors at the surface of endothelial cells cause an increased adhesiveness of platelets and leucocytes to the atrial endocardium, which initiates atrial thrombogenesis (*Graphical Abstract* and *Figure 1*). These alterations are not directly related to the presence of clinical or subclinical AF, and, therefore, help to explain why thrombogenesis and stroke risk are increased even during sinus rhythm.^5,35–41,48,49^

## 5. Screening for atrial cardiomyopathy

Despite being able to help to convey the primary underlying pathology in various clinical conditions, a number of issues limit the practical clinical application of a histopathological classification system for atrial cardiomyopathies.^5,38^ Therefore, alternative indicators of atrial cardiomyopathy by non-invasive investigations, such as imaging techniques, electrocardiography, and serum biomarkers, have been proposed,^5,35,38,41,50,55–60^ which may help in the future to define a tailored therapeutic approach in these patients.

Similar to frequent PACs, the presence of atrial activation impairment is also associated with a higher incidence of AF and stroke.^61–65^ Abnormal P-wave terminal force in lead V_1_ and increased P-wave duration have been associated with a higher risk of both AF and stroke.^61–65^ A higher P-wave maximal area have only been associated with a higher risk of stroke,^62^ and a prolonged PR interval, increased P-wave dispersion, and greater P-wave index have only been associated with a higher risk of AF.^61,63–65^ Nevertheless, despite being similar markers of the abnormal atrial substrate, their predictive capacity for AF or stroke seems to be lower than that of frequent PACs, especially when adjusted for confounding variables.^39,66^

Various circulating biomarkers have been associated with atrial cardiomyopathy, AF, and stroke.^40^ Recently, the PREDICT-AF study, conducted in a group of patients undergoing cardiac surgery, demonstrated that atrial remodelling can occur before incident AF and that the pattern of gene expression in the left atrium and the levels of correlated circulating biomarkers may be more predictive of incident AF than clinical parameters alone. However, these findings need confirmation in a broader patient population.^67^

Imaging markers suggestive of atrial cardiomyopathy include atrial dilatation, reduced left atrial (LA) strain during reservoir phase, diastolic dysfunction, LA spontaneous echo contrast, and reduced LA appendage emptying flow velocity, usually assessed by transthoracic and transoesophageal echocardiography, and direct identification of atrial fibrosis on cardiac magnetic ressonance.^5,35,55–60^

## 6. Imaging markers of the structural and functional changes related with frequent PACs

Currently, little information is available on the association of LA function and structure and frequent atrial ectopy.

In a recent prospective cohort study, using the Multi-Ethnic Study of Atherosclerosis (MESA) cohort of patients, LA function and dimensions were analysed using data from cardiovascular magnetic resonance imaging at the 2010–2012 examination, and PACs burden was analysed using data from 14-day ambulatory cardiac monitoring ∼6 years later, during the 2016–2018 examination.^68^ On multivariable analysis, greater LA total, passive, and active emptying fractions were each associated with fewer PACs/hour. Larger LA minimum volume was associated with more PACs/hour. Despite being associated with more PACs/hour in unadjusted analyses, lower peak LA longitudinal strain and larger LA maximum volume were not associated with a higher PAC occurrence after adjusting for confounding variables.^68^ The authors proposed that since LA enlargement and impaired LA function are associated with the development of incident AF and a higher risk of stroke independent of AF, these findings provide support for the hypothesis of a common atrial disease which promotes atrial ectopy, provides a substrate for AF, and may establish a thrombogenic environment.^68–71^

A smaller study by John *et al.*,^72^ based on 24 h Holter monitoring and transthoracic echocardiography imaging, found that patients with frequent PACs, defined as ≥100 PACs/24 h, exhibited lower LA peak contractile strain (−7.85 ± 4.12% vs. −9.33 ± 4.45%, *P* = 0.006) and a smaller LA peak late negative contractile strain rate (−0.63 ± 0.27 s^−1^ vs. −0.69 ± 0.32 s^−1^, *P* = 0.051) than the control group. LA volume index was also greater in patients with frequent PACs (26.6 ± 7.8 vs. 24.6 ± 8.8 mL/m^2^, *P* < 0.05). Another study by Py *et al.*, based on 48 h Holter monitoring and transthoracic and transoesophageal echocardiography imaging performed on patients after an acute ischaemic cerebrovascular accident, found that patients with frequent PACs, defined as >100 PACs/24 h, had higher LA minimal and maximal volumes (respectively, 17.8 ± 9 vs. 12.6 ± 6 mL/m^2^, *P* < 0.01, and 33.8 ± 10 vs. 28.8 ± 11 mL/m^2^, *P* < 0.01), and a lower LA total emptying fraction (48.6 ± 15 vs. 56.5 ± 11%, *P* < 0.01), even after adjusting for age and presence of hypertension. They also found that patients with excessive PACs had a lower LA appendage emptying velocity (66.9 ± 23.3 vs. 80.5 ± 28.4 cm/s, *P* = 0.003).^73^ Therefore, the authors concluded that frequent PACs after an acute ischaemic cerebrovascular accident are associated with LA myopathy, which might lead to cardiac embolism even in the absence of AF.^73^ They also highlighted the similarities of these findings with the abnormalities present in AF patients.^73–77^

## 7. The ideal definition of excessive PACs and its implications for treatment

Specific definitions of excessive atrial ectopic activity are lacking and the clinically important threshold value of PACs in predicting adverse outcomes is not known. At the present time, a 24 h Holter monitor is the gold standard for assessing PACs frequency.^1–4^

Many questions remain unanswered concerning the link between PACs, AF, stroke, and mortality. One is which patients should be referred for more stringent evaluation for AF upon detecting frequent PACs on Holter monitoring. Another is which patients with frequent PACs are most prone to poor outcomes. One more is whether treatment of patients with a high burden of PACs with antiarrhythmic medications or by means of catheter ablation could reduce the risk of developing AF, thereby reducing stroke risk and decreasing mortality. Finally, another important question is whether patients with an increased stroke risk profile who have a certain frequency of PACs could benefit from anticoagulation.^1–4^

In a recent consensus document by Arnar *et al.*,^4^ a high burden of PACs was defined as more than 500 PACs in 24 h. The reason for this chosen threshold was a study from Gladstone *et al.*^78^ [30-Day Cardiac Event Monitor Belt for Recording Atrial Fibrillation After a Cerebral Ischaemic Event (EMBRACE) Trial] in which the predicted probability of AF was 7–9% in patients with <100 PACs/24 h, 9–24% in those with 100–499 PACs/24 h, 25–37% in those with 500–999 PACs/24 h, 37–40% in those with 1000–1499 PACs/24 h, and it reached a plateau ≈40% in those with ≥1500 PACs/24 h.

Given the findings from EMBRACE trial^78^ and the possible mechanisms for the association of PACs with AF, stroke, and mortality that were previously discussed, Arnar *et al.*^4^ suggested the following:

1. Patients with a high PAC burden (>500/24 h) on Holter monitor should be considered at increased risk for developing of AF. Therefore, they should be educated on the symptoms of AF, and they should undergo further evaluation for possible AF including more detailed or prolonged rhythm monitoring;
2. Comprehensive cardiovascular risk factor modification is recommended for patients with a high PAC burden, and evaluation for structural heart disease should be considered in selected cases;
3. When brief episodes of AF are observed, the burden of PACs (>500 PACs/24 h or any episode of runs of more than 20 PACs) could add to the decision process whether anticoagulation therapy should be initiated;
4. Low-to-moderate PAC burden without documented AF is not an indication for oral anticoagulation.

However, the EMBRACE trial investigated the value of excessive PACs in predicting AF in patients with cryptogenic stroke, in whom previous subclinical AF episodes could have been related to the acute thromboembolic events.^78^ Therefore, >500 PACs/24 h is associated with a higher risk of AF after a cryptogenic stroke, but we still do not know if this threshold fits patients with PACs who have never experienced a stroke and do not have AF or other well-known risk factors for cerebrovascular events, in whom the increased risk of stroke could be directly related to atrial cardiomyopathy. Therefore, the framework suggested by Arnar *et al.*^4^ remains imperfect.

Moreover, since the majority of studies on the prognostic significance of PACs do not specify the type of ischaemic stroke that participants have suffered in terms of aetiology and since PACs have also been associated with traditional risk factors for cardiovascular disease, a comprehensive approach to these patients should also include intensive management of all cardiovascular risk factors and established vascular disease, such as carotid atherosclerosis or cerebral small-vessel disease, rather than just focusing on long-term rhythm monitoring and anticoagulation.^66^

Recently, Kaplan *et al.*^79^ evaluated the rate of stroke and systemic embolism as a function of both AF burden and stroke risk factors, represented by the ***C***ongestive Heart Failure, ***H***ypertension, ***A***ge ≥75 years, ***D***iabetes Mellitus, ***S***troke/Transient Ischaemic Attack/Thromboembolism, ***V***ascular Disease (prior myocardial infarction, peripheral arterial disease, or aortic plaque), ***A***ge 65 to 74 years, ***S***ex ***c***ategory (female) (CHA_2_DS_2_-VASc) score and found that patients with a CHA_2_DS_2_-VASc score ≥5 have a high risk of stroke and systemic embolism, between 1.7 and 2.2% per year, independently of AF occurrence, suggesting that some strokes may not be caused by AF directly but rather by other cardiovascular risk factors and mechanisms with which AF is frequently associated.

In a secondary analysis of the Rivaroxaban Versus Aspirin in Secondary Prevention of Stroke and Prevention of Systemic Embolism in Patients With Recent Embolic Stroke of Undetermined Source (ESUS) trial, which considered patients after an embolic stroke of undetermined source (ESUS) who were randomly assigned to receive either rivaroxaban 15 mg daily or aspirin 100 mg daily, an increasing frequency of PACs per 24 h was associated with an increasing risk of AF detection during follow-up, but the stroke risk was not reduced when patients received rivaroxaban compared with those who received aspirin. An increasing left atrial diameter also showed a trend to an increasing risk of AF detection, and patients with at least moderate atrial dilatation (i.e. with a left atrial diameter >46 mm) who were assigned to rivaroxaban had a 74% relative risk reduction of ischaemic stroke compared with patients who received aspirin. This benefit was not observed for those patients with smaller left atrial dimensions.^80^ However, the rivaroxaban dose used was lower than that recommended for stroke prevention in the majority of AF patients, i.e. 20 mg daily.^81^

Therefore, the frequency of PACs should be probably integrated with non-invasive investigations, namely echocardiography, to accurately identify those patients with a higher stroke risk more likely related to left atrial embolism and who could eventually benefit from an earlier anticoagulation strategy.^55,59,60^

The ongoing Atrial Cardiopathy and Antithrombotic Drugs In Prevention After Cryptogenic Stroke trial (NCT03192215 at clinicaltrials.gov) is attempting to address the role of anticoagulation for the prevention of recurrent stroke in patients with ESUS and evidence of atrial cardiomyopathy. It is expected to be completed by April 2022 and its results may bring some new insights about the management of patients with signs suggestive of atrial cardiomyopathy.^82^

In cases of ESUS, we should not forget that there are also other potential mechanisms that need to be investigated, like occult paroxysmal AF, patent foramen ovale, aortic arch atheroma, and atherosclerosis of the cervical or cerebral vessels of <50%, which may already have a clear indication for anticoagulation or may need an alternative therapeutic approach.^37,39,41,66,83^ Regarding the mechanisms implicated on AF initiation and maintenance, novel therapeutic priorities could include the identification of molecular targets at the level of ectopic beat generation, mechanisms leading to conduction abnormalities and gene-based pathways.^35,38,41,51^

## 8. Conclusion

Because of their documented association with traditional cardiovascular risk factors, atrial cardiomyopathy, AF, stroke, and death, frequent PACs should be no more considered a benign entity. A specific definition of excessive PACs is urgently needed as are prospective studies regarding antiarrhythmic therapies or anticoagulation in this group of patients. The effect of cardiovascular risk factors modification on the PAC burden should also be studied.

A histopathological classification system of atrial cardiomyopathies has limited applicability in clinical practice and alternative indicators of atrial cardiomyopathy by non-invasive investigations have been suggested to tailor the therapeutic approach in these patients. The PAC burden seems to be indicative of the atrial disease status, with later stages probably having more frequent PACs, requiring a more thorough investigation.

An effective management strategy for patients with excessive PACs must be holistic, encompassing not only rhythm monitoring and non-invasive investigations for atrial cardiomyopathy, with eventual anticoagulation according to CHA_2_DS_2_-VASc score, especially if AF is detected, but also the tight control of cardiovascular risk factors and the treatment of established vascular disease.

Novel therapeutic priorities could also include the identification of molecular targets at the level of ectopic beat generation, mechanisms leading to conduction abnormalities and gene-based pathways that are implicated in AF initiation and maintenance.

## Supplementary Material

cvac054_Supplementary_Data
