# Supplementary material for: Frequent premature atrial contractions as a signalling marker of atrial cardiomyopathy, incident atrial fibrillation, and stroke
Source: Cardiovasc Res. 2022 Apr 7;119(2):429–39. doi: 10.1093/cvr/cvac054 (PMC10064848; doi:10.1093/cvr/cvac054)
Supplement: cvac054_Supplementary_Data [file cvac054_supplementary_data.zip › Supplementary Material - Search Strategy.docx]

**SUPPLEMENTAL MATERIAL**

**SEARCH STRATEGY**

We searched the PubMed database for relevant reviews and original research studies.

Regarding the association of PACs with incident AF, stroke or mortality, we used the following keywords in the search strategy: (premature atrial contractions or premature atrial complexes or supraventricular ectopic beats or excessive supraventricular ectopic activity or atrial ectopy) and (cardiovascular outcomes or atrial fibrillation or stroke or brain ischemia or mortality or death).

Regarding the electrophysiological mechanisms of PACs, we searched for reviews on “premature atrial contractions”, “premature atrial complexes”, and “atrial ectopy”. For the electrophysiological mechanisms of AF, we used the following search strategy: (atrial fibrillation) and (mechanisms or molecular basis).

Regarding the pathophysiology of atrial cardiomyopathy, we used the following keywords in the search strategy: “atrial cardiopathy”, “atrial cardiomyopathy”, “atrial myopathy”, and “atrial fibrosis”. For thrombogenic mechanisms in AF, we used the following keywords: (atrial fibrillation) and (thrombogenesis or prothrombotic state or Virchow triad).

Regarding the atrial imaging in patients with frequent PACs, we conducted a search using the following keywords: (premature atrial contractions or premature atrial complexes or supraventricular ectopic beats or excessive supraventricular ectopic activity or atrial ectopy) and (atrial fibrosis or cardiac magnetic resonance or echocardiography or imaging or left atrial dimensions or left atrial function).

Regarding the question about anticoagulant treatment of PACs, we used the following search strategy: (premature atrial contractions or premature atrial complexes or supraventricular ectopic beats or excessive supraventricular ectopic activity or atrial ectopy) and (anticoagulation or anticoagulant treatment or anticoagulant therapy). Using the same keywords, we also searched directly on the official Journal of the European Heart Rhythm Association (EHRA) – the EP Europace Journal – for any consensus document about the use of anticoagulation in PACs.
