# Supplementary material for: Frequent premature atrial contractions as a signalling marker of atrial cardiomyopathy, incident atrial fibrillation, and stroke
Source: Cardiovasc Res. 2022 Apr 7;119(2):429–39. doi: 10.1093/cvr/cvac054 (PMC10064848; doi:10.1093/cvr/cvac054)
Supplement: cvac054_Supplementary_Data [file cvac054_supplementary_data.zip › Supplementary Material - Supplementary Table 1.docx]

SUPPLEMENTARY TABLE 1. Studies on frequent PACs and their association with incident atrial fibrillation included in the meta-analyses presented in TABLE 1

| Author, year | Study design | Total number of patients | Age, in years | Male gender, in % | Baseline recording device | Follow-up, in years | Definition of PAC-count as the predictor | Effect measure (95% CI) of the association between PAC-count and AF | Incidence rate of AF, in absolute frequency (%) and per 1,000 PYs |
| --- | --- | --- | --- | --- | --- | --- | --- | --- | --- |
| Binici  2010^7^ | P | 678 | 64.5 ± 6.8 | 58.6 | 48-h Holter | 6.3 (6.2 – 6.5) | ≥30 PACs/h or any runs of ≥20 PACs (ESVEA) (Dic) | UV HR 3.19 (1.30-7.86)  MV HR 2.73 (1.07-6.96) | **Total cohort:**  22/678 (3.2%); 5.5/1,000 PYs  **ESVEA group:**  7/99 (7.1%); 12.8/1,000 PYs  **Non-ESVEA group:**  15/579 (2.6%); 4.3/1,000 PYs |
|  |  |  |  |  |  |  | PACs/h (Con, linear for each increment of 10 PACs/h) | UV HR 1.57 (1.12-2.21)  MV 1.49 (1.02-2.17) |  |
|  |  |  |  |  |  |  | Length of runs of PACs (Con, linear for lengthening of run by every 4 PACs) | UV HR 1.30 (1.15-1.47)  MV HR 1.29 (1.14-1.47) |  |
| Marinheiro  2017^10^ | P | 362 | 71.3 ± 7.8 | 56.4 | 24-h Holter | Median of 7.1 | >97 PACs/h (Dic) | UV HR 1.87 (1.24-2.81)  MV HR 1.76 (1.17-2.66)‡ | **Total cohort:**  39/362 (10.8%)  **>97 PACs/h group:**  67.2/1,000 PYs  **30-97 PACs/h group:**  55.8/1,000 PYs  **<30 PACs/h group:**  33.3/1,000 PYs |
|  |  |  |  |  |  |  | PACs/h (Con, LT) | UV HR 1.44 (1.14-1.81)  MV HR 1.40 (1.15-1.72)‡ |  |
|  |  |  |  |  |  |  | >97 PACs/h vs. <30 PACs/h (Ord) | UV HR 2.05 (1.31-3.20)  MV HR 2.05 (1.31–3.23) |  |
| Chong  2012^11^ | P | 428 | 66.7 ± 10.2 | 43.7 | 24-h Holter | 6.1 ± 1.3 | >100 PACs/24h (Dic) | UV HR 3.9 (3.2-11.1)  MV HR 3.22 (1.9-5.5) | **Total cohort:**  60/428 (14.0%)  **>100 PACs/24h group:**  31/107 (29.0%); 48.3/1,000 PYs  **≤100 PACs/24h group:**  29/321 (9.0%); 14.3/1,000 PYs |
| Acharya  2015^12^ | R | 1,357 | Mean of 64.0 | 93.0 | 24-h Holter | Median of 7.5 | ≥100 PACs/24h (Dic) | UV HR 4.34 (3.09-6.08)  MV HR 2.97 (1.85-4.80) | **Total cohort:**  155/1357 (11.4%)  **≥100 PACs/24h group:**  106/486 (21.8%)  **<100 PACs/24h group:**  49/871 (5.6%) |
| Lin  2015^13^ | R | 5,371 | 61.8 ± 18.6 | 60.0 | 24-h Holter | 10.0 ± 1.0 | >76 PACs/24h (Dic) | UV HR 2.31 (1.90-2.80)  MV HR 1.76 (1.43-2.16) | **Total cohort:**  418/5,371 (7.8%)  **>76 PACs/24h group:**  242/2,072 (11.7%)  **≤76 PACs/24h group:**  176/3,299 (5.3%) |
| Suzuki  2013^19^ | R | 2,589 | 54.2 ± 15.5 | 55.5 | 24-h Holter | 1.6 ± 1.7 | ≥102 PACs/24h (Dic) | UV HR 9.44 (4.85-18.37)*  MV HR 6.89 (3.45-13.74)* | **Total cohort:**  38/2589 (1.5%)  **≥102 PACs/24h group:**  27/647 (3.4%); 23.8/1,000 PYs  **<102 PACs/24h group:**  11/1942 (0.6%); 15.5/1,000 PYs |
| Vinther  2016^20^ | R | 565 | Mean of 71.5 | 55.4 | 48-h Holter | Mean of 4.0 | Runs of ≥3 PACs (Dic) | UV HR 1.44 (0.61-3.44)  MV HR 1.24 (0.50-3.09) | **Total cohort:**  22/565 (3.9%)  **With runs of PACs group:**  8/161 (5.0%)  **Without runs of PACs group:**  14/404 (3.5%) |
| Vinther  2017^21^ | P | 167 | Mean of 69.9 | 60.5 | 24-h Holter | Median of 2.7 | >14 PACs/h and ≥3 runs of ≥3 consecutive PACs/24h (Dic) | UV HR 3.55 (0.98-12.8)**  MV HR 3.05 (0. 70-13.3)** | **Total cohort:**  9/167 (5.4%)  **AF in “frequent PACs” and “infrequent PACs” groups:**  N/A |
|  |  |  |  |  |  |  | PACs/24h (Con, LT) | UV HR 1.29 (1.04-1.60)**  MV HR 1.24 (0.89-1.73)** |  |
|  |  |  |  |  |  |  | Runs of ≥3 PACs (Dic) | UV HR 2.18 (0.57-8.36)**  MV HR 1.24 (0.27-5.63)** |  |
| Yamada  2000^22^ | P | 75 | 65 ± 11 | 81.3 | 24-h Holter | 1.8 ± 0.8 | >100 PACs/h or any runs of ≥ 2 PACs (Dic) | UV HR 3.7 (1.0-13.2)  MV HR 1.1 (0.2-6.3) | **Total cohort:**  10/75 (13.3%)  **>100 PACs/h or any runs of ≥ 2 PACs group:**  6/23 (26.1%)  **≤100 PACs/h and without runs of PACs group:**  4/52 (7.7%) |
| Yodogawa  2013^23^ | P | 68 | 69.9 ± 9.6 | 54.4 | 24-h Holter | 0.9 ± 0.3 | >100 PACs/24h (Dic) | UV HR N/A  MV HR 4.53 (1.5-13.6) | **Total cohort:**  17/68 (25.0%)  **AF in “frequent PACs” and “infrequent PACs” groups:**  N/A |
| Cabrera 2016^14^ | R | 299 | 62.5 ± 17.9 | 53.5 | 24-h Holter | 3.3 (3.1-3.3) | ≥0.2% PACs/24h (Dic) | UV HR 3.64 (1.78-7.4)  MV HR 2.7 (1.2-5.8) | **Total cohort:**  31/299 (10.4%)  **AF in “frequent PACs” and “infrequent PACs” groups:**  N/A |
| Johnson 2015^15^ | P | 383 | 64.6 ± 5.9 | 44.9 | 24-h Holter | Mean of 10.3 | ≥30 PACs/h or any runs of ≥20 PACs (Dic) | UV HR 3.91 (2.12-7.21)‡  MV HR 2.66 (1.38-5.12)** | **Total cohort:**  45/383 (11.7%)  **AF in “frequent PACs” and “infrequent PACs” groups:**  N/A |
|  |  |  |  |  |  |  | PACs/h (Con, LT) | UV HR N/A  MV HR 1.39 (1.16-1.68)** |  |
|  |  |  |  |  |  |  | Number of runs of ≥3 PACs/h (Con, LT) | UV HR N/A  MV HR 1.99 (1.40-2.82)** |  |
| Nortamo  2017^16^ | P | 1,710 | Mean of 66.5 | N/A | 24-h Holter | 5.6 ± 1.5 | Any run of ≥4 PACs <30 sec (SVR) (Dic) | UV HR 2.60 (1.83-3.69)  MV HR 2.53 (1.76-3.63) | **Total cohort:**  143/1,710 (8.4%)  **SVR group:**  47/275 (17%)  **Non-SVR group:**  96/1,435 (7%) |
|  |  |  |  |  |  |  | ≥1,427 PACs/24h vs. <507 PACs/24h (Ord) | UV HR 8.25 (4.13-16.48)  MV HR 8.14 (3.97-16.70) |  |
| Pinho  2015^17^ | R | 205# | 55.2 ± 15.1 | 52.2 | 24-h Holter | 2.3 (1.0-4.6) | >30 PACs/h (Dic) | UV HR 4.04 (1.43-11.40)‡  HR MV 2.28 (0.69-7.55)‡ | **Total cohort:**  21/205 (10.2%)  **AF in “frequent PACs” and “infrequent PACs” groups:**  N/A |
|  |  |  |  |  |  |  | PACs/h (Con, LT) | UV HR 1.92 (1.29-2.87)‡  MV HR 1.44 (0.92-2.25)‡ |  |
| Raman 2017^18^ | P | 2,350 | 75.8 ± 5.3 | 100.0 | PSG ECG | 8.0 ± 2.6 | ≥21.15 PACs/h (Dic) | UV HR 1.77 (1.38-2.28)‡  MV HR 1.53 (1.18-1.98)‡ | **Total cohort:**  269/2,350 (11.4%)  **≥5 PACs/h group:**  185/1278 (14.5%)  **<5 PACs/h group:**  84/1072 (7.8%) |
|  |  |  |  |  |  |  | ≥21.15 PACs/h vs. <2.19 PACs/h (Ord) | UV HR 3.56 (2.35-5.39)  MV HR 2.99 (1.94-4.62) |  |
|  |  |  |  |  |  |  | PACs/h (Con, LT) | UV HR 1.58 (1.35-1.84)‡  MV HR 1.44 (1.23-1.70)‡ |  |
|  |  |  |  |  |  |  | ≥5 PACs/h (Dic) | UV HR N/A  MV HR N/A |  |
| Dewland 2013^24^ | P | 1,260 | 71 (68-75) | 45.2 | 24-h Holter | 13.0 (7.3-18.1) | PACs/h (Con, LT) | UV HR 1.18 (1.14-1.22)**  MV HR 1.17 (1.13-1.22)** | **Total cohort:**  343/1260 (27.2%)  **AF in “frequent PACs” and “infrequent PACs” groups:**  N/A |
|  |  |  |  |  |  |  | ≥9.5 PACs/h vs. <0.8PACs/h (Ord) | UV HR 5.01 (3.50-7.17)**  MV HR 4.92 (3.39-7.16)** |  |
| Thijs  2016^25^ | P | 221 | 61.6 ± 11.4 | 64.3 | 24-h Holter | 1.7 ± 0.8 | >123 PACs/24h vs. 0 PACs/24h (Ord) | At 12 months:  UV HR 3.94 (1.30-11.97)  MV HR N/A  At 36 months:  UV HR 3.47 (1.38-8.70)  MV HR N/A | **Total cohort at 36 months:**  42/221 (19.0%)  **AF in “frequent PACs” and “infrequent PACs” groups:**  N/A |

AF – atrial fibrillation; Con – continuous; Dic – dichotomous; ESVEA – excessive supraventricular ectopic activity; LT – log-transformed; MV – multivariate (adjusted); N/A = not available; Ord – ordinal; P – prospective; PAC(s) – premature atrial contraction(s); PSG ECG – polysomnography continuous electrocardiogram; PYs – person-years; R – retrospective; SVR - supraventricular run; UV – univariate (unadjusted);

* These values are the result of a meta-analysis performed by Himmelreich *et al.*^2^ of separate results presented by Suzuki *et al.*^19^

** Death as a competing risk

# The original report included only 184 patients. Pinho *et al.*^17^ later provided Himmelreich *et al.*^2^ with data based on all of the 205 participants.

‡ Previously unpublished data provided by the authors to Himmelreich *et al.*^2^
