# Supplementary material for: Frequent premature atrial contractions as a signalling marker of atrial cardiomyopathy, incident atrial fibrillation, and stroke
Source: Cardiovasc Res. 2022 Apr 7;119(2):429–39. doi: 10.1093/cvr/cvac054 (PMC10064848; doi:10.1093/cvr/cvac054)
Supplement: cvac054_Supplementary_Data [file cvac054_supplementary_data.zip › Supplementary Material - Supplementary Table 2.docx]

SUPPLEMENTARY TABLE 2. Studies on frequent PACs and their association with stroke included in the meta-analyses presented in TABLE 1

| Author, year | Study design | Total number of patients | Age, in years | Male gender, in % | Baseline recording device | Follow-up, in years | Definition of PAC-count as the predictor | Effect measure (95% CI) of the association between PAC-count and stroke | Incidence rate of stroke, in absolute frequency (%) and per 1,000 PYs |
| --- | --- | --- | --- | --- | --- | --- | --- | --- | --- |
| Binici  2010^7^ | P | 678 | 64.5 ± 6.8 | 58.6 | 48-h Holter | 6.3 (6.2 – 6.5) | ≥30 PACs/h or any runs of ≥20 PACs (ESVEA) (Dic) | UV HR 3.88 (1.78-8.48)  MV HR 2.37 (1.02-5.50) | **Total cohort:**  27/678 (4.0%); 6.7/1,000 PYs  **ESVEA group:**  10/99 (10.1%) 18.8/1,000 PYs  **Non-ESVEA group:**  17/579 (2.9%) 4.9/1,000 PYs |
|  |  |  |  |  |  |  | PACs/h (Con, linear for each increment of 10 PACs/h) | UV HR 0.83 (0.58-1.98)  MV HR N/A |  |
|  |  |  |  |  |  |  | Length of runs of PACs (Con, linear for lengthening of run by every 4 PACs) | UV HR 1.12 (0.97-1.29)  MV HR N/A |  |
| Engström  2000^8^ | P | 388 | 68 | 100.0 | 24-h Holter | 10.6 ± 4.2 | ≥218 PACs/24h (Dic) | UV RR N/A  MV RR 1.90 (1.02-3.40) | **Total cohort:**  54/388 (13.9%)  **≥218 PACs/24h group:**  15/77 (19.5%); 19.5/1,000 PYs  **<218 PACs/24h group:**  39/311 (12.5%); 11.6/1,000 PYs |
| Marinheiro  2017^10^ | P | 362 | 71.3 ± 7.8 | 56.4 | 24-h Holter | Median of 7.1 | >97 PACs/h (Dic) | UV HR 2.17 (1.12-4.19)‡  MV HR 2.01 (1.03-3.93)‡ | **Total cohort:**  54/362 (14.9%)  **>97 PACs/h group:**  34.9/1,000 PYs  **30-97 PACs/h group:**  15.1/1,000 PYs  **<30 PACs/h group:**  11.5/1,000 PYs |
|  |  |  |  |  |  |  | PACs/h (Con, LT) | UV HR 1.66 (1.18-2.35)‡  MV HR 1.71 (1.21-2.43)‡ |  |
|  |  |  |  |  |  |  | >97 PACs/h vs. <30 PACs/h (Ord) | UV HR 2.71 (1.60-4.61)  MV HR 2.83 (1.65–4.84) |  |
| Chong  2012^11^ | P | 428 | 66.7 ± 10.2 | 43.7 | 24-h Holter | 6.1 ± 1.3 | >100 PACs/24h (Dic) | UV HR 2.1 (1.1-4.8)  MV HR N/A | **Total cohort:**  41/428 (9.6%); 15.4/1,000 PYs  **>100 PACs/24h group:**  16/107 (15.0%)  **≤100 PACs/24h group:**  25/321 (7.8%) |
| Lin  2015^13^ | R | 5,371 | 61.8 ± 18.6 | 60.0 | 24-h Holter | 10.0 ± 1.0 | >76 PACs/24h (Dic) | UV RR 2.50 (0.97-6.44)†⁂  MV RR N/A | **Total cohort:**  18/5,371 (0.3%)⁂  **>76 PACs/24h group:**  11/2,072 (0.5%)⁂  **≤76 PACs/24h group:**  7/3,299 (0.2%)⁂ |
| Inohara  2013^26^ | P | 7,692 | 52.5 ± 13.7 | 41.5 | 12-lead ECG | 14.0 ± 2.9 | ≥1 PAC (Dic) | UV RR 4.48 (1.90-10.57)†⁂  MV RR N/A | **Total cohort:**  138/7,692 (1.8%)⁂  **≥1 PAC group:**  5/64 (7.8%)⁂  **No PACs group:**  133/7,628 (1.7%)⁂ |
| Larsen  2015^27^ | P | 678 | 64.5 ± 6.8 | 58.6 | 48-h Holter | Median of 14.4 | ≥30 PACs/h or any runs of ≥20 PACs (ESVEA) (Dic) | UV HR 2.07 (1.21-3.56)  MV HR 2.02 (1.17-3.49) | **Total cohort:**  73/678 (10.8%)  **ESVEA group:**  21.5/1,000 PYs  **Non-ESVEA group:**  7.4/1,000 PYs |
|  |  |  |  |  |  |  | ≥30 PACs/h (Dic) | UV HR 2.24 (1.23-4.09)  MV HR 1.60 (0.86-3.02) |  |
|  |  |  |  |  |  |  | Any runs of ≥20 PACs (Dic) | UV HR 2.82 (1.44-5.50)  MV HR 2.26 (1.10-4.64) |  |
| Murakoshi  2015^28^ | P | 63,197 | 58.8 ± 9.9 | 32.4 | 15-s ECG | Mean of 14.3 | ≥1 PAC (Dic) | Men  UV HR 2.32 (1.85-2.91)⁂  MV HR 1.24 (0.98-1.56)⁂  Women  UV HR 3.10 (2.48-3.87)⁂  MV HR 1.63 (1.30-2.05)⁂ | **Total cohort:**  1208/63,197 (1.9%)⁂  Men: 563/20,492 (2.7%)⁂  Women: 645/42,705 (1.5%)⁂  **≥1 PAC group at 10 years:**  Total: 106/3,858 (2.7%)⁂  Men: 64/1,650 (3.9%)⁂  Women: 42/2,208 (1.9%)⁂  **No PACs group at 10 years:**  Total: 507/59,339 (0.9%)⁂  Men: 264/18,842 (1.4%)⁂  Women: 243/40,497 (0.6%)⁂ |
| Ofoma  2012^29^ | P | 14,493 | Mean of 54.0 | 43.3 | 2-min ECG | Mean of 13 | ≥1 PAC (Dic) | UV HR 1.68 (1.21-2.32)  MV HR 1.30 (0.92-1.83) | **Total cohort:**  509/14,493 (3.5%)  **≥1 PAC group:**  39/717 (5.4%)  **No PACs group:**  470/13,776 (3.4) |
| O’Neal  2016^30^ | P | 22,975 | 64.0 ± 9.2 | 44.0 | 12-lead ECG | Up to 11 | ≥1 PAC (Dic) | UV HR N/A  MV HR 1.34 (1.04-1.74) | **Total cohort:**  549/22,975 (2.4%)  **≥1 PAC group:**  68/1,687 (4.0%); 6.0/1,000 PYs  **No PACs group:**  481/21,288 (2.3%); 3.2/1,000 PYs |

Con – continuous; Dic – dichotomous; ESVEA – excessive supraventricular ectopic activity; LT – log-transformed; MV – multivariate (adjusted); N/A = not available; Ord – ordinal; P – prospective; PAC(s) – premature atrial contraction(s); PYs – person-years; R – retrospective; SVR - supraventricular run; UV – univariate (unadjusted)

‡ Previously unpublished data provided by the authors to Himmelreich *et al.*^2^

†Values obtained by Huang *et al.*^3^

⁂ Refer only to fatal stroke
