# Supplementary material for: Frequent premature atrial contractions as a signalling marker of atrial cardiomyopathy, incident atrial fibrillation, and stroke
Source: Cardiovasc Res. 2022 Apr 7;119(2):429–39. doi: 10.1093/cvr/cvac054 (PMC10064848; doi:10.1093/cvr/cvac054)
Supplement: cvac054_Supplementary_Data [file cvac054_supplementary_data.zip › Supplementary Material - Supplementary Table 3.docx]

SUPPLEMENTARY TABLE 3. Studies on frequent PACs and their association with all-cause mortality included in the meta-analyses presented in TABLE 1

| Author, year | Study design | Total number of patients | Age, in years | Male gender, in % | Baseline recording device | Follow-up, in years | Definition of PAC-count as the predictor | Effect measure (95% CI) of the association between PAC-count and ACM | Incidence rate of ACM, in absolute frequency (%) and per 1,000 PYs |
| --- | --- | --- | --- | --- | --- | --- | --- | --- | --- |
| Binici  2010^7^ | P | 678 | 64.5 ± 6.8 | 58.6 | 48-h Holter | 6.3 (6.2 – 6.5) | ≥30 PACs/h or any runs of ≥20 PACs (ESVEA) (Dic) | UV HR 2.12 (1.30-3.47)  MV HR 1.40 (0.83-2.36) | **Total cohort:**  87/678 (12.8%); 21.4/1,000 PYs  **ESVEA group:**  21/99 (21.2%); 37.2/1,000 PYs  **Non-ESVEA group:**  66/579 (11.4%); 18.9/1,000 PYs |
|  |  |  |  |  |  |  | PACs/h (Con, linear for each increment of 10 PACs/h) | UV HR 1.49 (1.24-1.79)  MV HR 1.27 (1.04-1.55) |  |
|  |  |  |  |  |  |  | Length of runs of PACs (Con, linear for lengthening of run by every 4 PACs) | UV HR 1.12 (1.03-1.21)  MV HR 1.06 (0.97-1.15) |  |
| Engström  2000^8^ | P | 388 | 68 | 100.0 | 24-h Holter | 10.6 ± 4.2 | ≥218 PACs/24h (Dic) | UV RR N/A  MV RR 1.3 (0.88-1.80) | **Total cohort:**  170/388 (43.8%)  **≥218 PACs/24h group:**  40/77 (51.9%); 48.3/1,000 PYs  **<218 PACs/24h group:**  130/311 (41.8%); 37.1/1,000 PYs |
| Marinheiro  2017^10^ | P | 362 | 71.3 ± 7.8 | 56.4 | 24-h Holter | Median of 7.1 | >97 PACs/h (Dic) | UV HR 2.04 (1.35-3.08)‡  MV HR 1.84 (1.21-2.81)‡ | **Total cohort:**  129/362 (35.6%)  **>97 PACs/h group:**  77.8/1,000 PYs  **30-97 PACs/h group:**  56.8/1,000 PYs  **<30 PACs/h group:**  33.3/1,000 PYs |
|  |  |  |  |  |  |  | PACs/h (Con, LT) | UV HR 1.38 (1.11-1.71)‡  MV HR 1.38 (1.11-171)‡ |  |
|  |  |  |  |  |  |  | >97 PACs/h vs. <30 PACs/h (Ord) | UV HR 2.40 (1.54-3.73)  MV HR 2.17 (1.48-3.28) |  |
| Chong  2012^11^ | P | 428 | 66.7 ± 10.2 | 43.7 | 24-h Holter | 6.1 ± 1.3 | >100 PACs/24h (Dic) | UV HR 1.8 (1.1-3.6)  MV HR N/A | **Total cohort:**  69/428 (16.1%)  **>100 PACs/24h group:**  24/107 (22.4%)  **≤100 PACs/24h group:**  45/321 (14.0%) |
| Lin  2015^13^ | R | 5,371 | 61.8 ± 18.6 | 60.0 | 24-h Holter | 10.0 ± 1.0 | >76 PACs/24h (Dic) | UV HR 2.19 (1.95-2.45)  MV HR 1.38 (1.23-1.59) | **Total cohort:**  1,209/5,371 (22.5%)  **>76 PACs/24h group:**  671/2,072 (32.4%)  **≤76 PACs/24h group:**  538/3,299 (16.3%) |
| Vinther  2016^15^ | R | 565 | Mean of 71.5 | 55.4 | 48-h Holter | Mean of 4.0 | Runs of ≥3 PACs (Dic) | UV HR 1.79 (1.30-2.47)  MV HR 1.39 (0.99-1.96) | **Total cohort:**  158/565 (28.0%)  **ACM in “frequent PACs” and “infrequent PACs” groups:**  N/A |
| Vinther  2017^16^ | P | 167 | Mean of 69.9 | 60.5 | 24-h Holter | Median of 2.7 | >14 PACs/h and ≥3 runs of ≥3 consecutive PACs/24h (Dic) | UV HR 3.84 (1.94-7.61)‡  MV HR 2.06 (0.97-4.35) | **Total cohort:**  34/167 (20.4%)  **ACM in “frequent PACs” and “infrequent PACs” groups:**  N/A |
|  |  |  |  |  |  |  | PACs/24h (Con, LT) | UV HR 1.33 (1.16-1.53)‡  MV HR 1.14 (0.96-1.34) |  |
|  |  |  |  |  |  |  | Runs of ≥3 PACs (Dic) | UV HR 2.81 (1.34-5.87)  MV HR 1.85 (0.87-3.92) |  |
| Dewland 2013^24^ | P | 1,260 | 71 (68-75) | 45.2 | 24-h Holter | 13.0 (7.3-18.1) | PACs/h (Con, LT) | UV HR 1.10 (1.07-1.13)  MV HR 1.06 (1.03-1.09) | **Total cohort:**  837/1,260 (66.4%); 573/1,260 (45.5%) died without known AF  **ACM in “frequent PACs” and “infrequent PACs” groups:**  N/A |
|  |  |  |  |  |  |  | ≥9.5 PACs/h vs. <0.8PACs/h (Ord) | UV HR 1.93 (1.59-2.34)  MV HR 1.35 (1.10-1.66) |  |
| Inohara  2013^26^ | P | 7,692 | 52.5 ± 13.7 | 41.5 | 12-lead ECG | 14.0 ± 2.9 | ≥1 PAC (Dic) | UV HR 3.98 (2.77-5.71)  MV HR 1.55 (1.07-2.24) | **Total cohort:**  1,211/7,692 (15.7%)  **≥1 PAC group:**  30/64 (46.9%)  **No PACs group:**  1,181/7,628 (15.5%) |
| Murakoshi  2015^28^ | P | 63,197 | 58.8 ± 9.9 | 32.4 | 15-s ECG | Mean of 14.3 | ≥1 PAC (Dic) | UV HR 1.93 (1.70-2.20)●  MV HR 1.14 (1.02-1.27)● | **Total cohort at 10 years:**  4,178/63,197 (6.6%)  **≥1 PAC group at 10 years:**  483/3,858 (12.5%)  **No PACs group at 10 years:**  3,695/59,339 (6.2%) |
| Qureshi  2014^31^ | P | 7,394 | Mean of 59.2 | 53.1 | 10-s ECG | 13 ± 4 | ≥1 PAC (Dic) | UV HR 3.38 (2.67-4.27)  MV HR 1.41 (1.08-1.80) | **Total cohort:**  2,458/7,394 (33.2%)  **≥1 PAC group:**  72/89 (80.9%); 0.858/1,000 PYs  **No PACs group:**  2,386/7,305 (32.7%); 0.254/1,000 PYs |

ACM – all-cause mortality; Con – continuous; Dic – dichotomous; ESVEA – excessive supraventricular ectopic activity; LT – log-transformed; MV – multivariate (adjusted); N/A = not available; Ord – ordinal; P – prospective; PAC(s) – premature atrial contraction(s); PYs – person-years; R – retrospective; UV – univariate (unadjusted)

‡ Previously unpublished data provided by the authors to Himmelreich *et al.*^2^

● These values are the result of a meta-analysis performed by Himmelreich *et al.*^2^ of separate results presented by Murakoshi *et al.*^28^
