# Supplementary material for: Frequent premature atrial contractions as a signalling marker of atrial cardiomyopathy, incident atrial fibrillation, and stroke
Source: Cardiovasc Res. 2022 Apr 7;119(2):429–39. doi: 10.1093/cvr/cvac054 (PMC10064848; doi:10.1093/cvr/cvac054)
Supplement: cvac054_Supplementary_Data [file cvac054_supplementary_data.zip › Supplementary Material - Supplementary Table 4.docx]

SUPPLEMENTARY TABLE 4. Studies on frequent PACs and their association with cardiovascular death included in the meta-analyses presented in TABLE 1

| Author, year | Study design | Total number of patients | Age, in years | Male gender, in % | Baseline recording device | Follow-up, in years | Definition of PAC-count as the predictor | Effect measure (95% CI) of the association between PAC-count and CVD | Incidence rate of CVD, in absolute frequency (%) and per 1,000 PYs |
| --- | --- | --- | --- | --- | --- | --- | --- | --- | --- |
| Lin  2015^13^ | R | 5,371 | 61.8 ± 18.6 | 60.0 | 24-h Holter | 10.0 ± 1.0 | >76 PACs/24h (Dic) | UV RR 2.00 (1.60-2.50)†  MV RR N/A | **Total cohort:**  291/5,371 (5.4%)  **>76 PACs/24h group:**  162/2,072 (7.8%)  **≤76 PACs/24h group:**  129/3,299 (3.9%) |
| Inohara  2013^26^ | P | 7,692 | 52.5 ± 13.7 | 41.5 | 12-lead ECG | 14.0 ± 2.9 | ≥1 PAC (Dic) | UV HR 5.79 (3.25-10.30)  MV HR 2.03 (1.12-3.66) | **Total cohort:**  338/7,692 (4.4%)  **≥1 PAC group:**  12/64 (18.8%)  **No PACs group:**  326/7,628 (4.3%) |
| Murakoshi  2015^28^ | P | 63,197 | 58.8 ± 9.9 | 32.4 | 15-s ECG | Mean of 14.3 | ≥1 PAC (Dic) | Men  UV HR 2.18 (1.86-2.55)  MV HR 1.22 (1.04-1.44)  Women  UV HR 2.77 (2.35-3.26)  MV HR 1.48 (1.25-1.74) | **Total cohort:**  2,527/63,197 (4.0%)  Men: 1,205/20,492 (5.9%)  Women: 1,322/42,705 (3.1%)  **≥1 PAC group at 10 years:**  Total: 190/3,858 (4.9%)  Men: 111/1,650 (6.7%)  Women: 79/2,208 (3.6%)  **No PACs group at 10 years:**  Total: 1,014/59,339 (1.7%)  Men: 528/18,842 (2.8%)  Women: 486/40,497 (1.2%) |
| Qureshi  2014^31^ | P | 7,394 | Mean of 59.2 | 53.1 | 10-s ECG | 13 ± 4 | ≥1 PAC (Dic) | UV HR 4.27 (3.08-5.94)  MV HR 1.64 (1.13-2.36) | **Total cohort:**  1,000/7,394 (13.5%)  **≥1 PAC group:**  37/89 (41.6%); 0.441/1,000 PYs  **No PACs group:**  963/7,305 (13.2%); 0.103/1,000 PYs |

CVD – cardiovascular death; Dic – dichotomous; MV – multivariate (adjusted); N/A = not available; P – prospective; PAC(s) – premature atrial contraction(s); PYs – person-years; R – retrospective; UV – univariate (unadjusted)

† Values obtained by Huang *et al.*^3^
