# Supplementary material for: Frequent premature atrial contractions as a signalling marker of atrial cardiomyopathy, incident atrial fibrillation, and stroke
Source: Cardiovasc Res. 2022 Apr 7;119(2):429–39. doi: 10.1093/cvr/cvac054 (PMC10064848; doi:10.1093/cvr/cvac054)
Supplement: cvac054_Supplementary_Data [file cvac054_supplementary_data.zip › Supplementary Material - Supplementary Table 5.docx]

SUPPLEMENTARY TABLE 5. Studies on frequent PACs and their association with death from coronary artery disease included in the meta-analyses presented in TABLE 1

| Author, year | Study design | Total number of patients | Age, in years | Male gender, in % | Baseline recording device | Follow-up, in years | Definition of PAC-count as the predictor | Effect measure (95% CI) of the association between PAC-count and DCAD | Incidence rate of DCAD, in absolute frequency (%) and per 1,000 PYs |
| --- | --- | --- | --- | --- | --- | --- | --- | --- | --- |
| Lin  2015^13^ | R | 5,371 | 61.8 ± 18.6 | 60.0 | 24-h Holter | 10.0 ± 1.0 | >76 PACs/24h (Dic) | UV RR 1.91 (1.18-3.09)†  MV RR N/A | **Total cohort:**  66/5,371 (1.2%)  **>76 PACs/24h group:**  36/2,072 (1.7%)  **≤76 PACs/24h group:**  30/3,299 (0.9%) |
| Inohara  2013^26^ | P | 7,692 | 52.5 ± 13.7 | 41.5 | 12-lead ECG | 14.0 ± 2.9 | ≥1 PAC (Dic) | UV RR 1.78 (0.25-12.62)†  MV RR N/A | **Total cohort:**  68/7,692 (0.9%)  **≥1 PAC group:**  1/64 (1.6%)  **No PACs group:**  67/7,628 (0.9%) |
| Qureshi  2014^31^ | P | 7,394 | Mean of 59.2 | 53.1 | 10-s ECG | 13 ± 4 | ≥1 PAC (Dic) | UV HR 5.84 (3.97-8.61)  MV HR 2.06 (1.28-3.13) | **Total cohort:**  538/7,394 (7.3%)  **≥1 PAC group:**  27/89 (30.3%); 0.321/1,000 PYs  **No PACs group:**  511/7,305 (7.0%); 0.055/1,000 PYs |
| Algra  1993^32^ | R | 6,693 | ≥60: 50.3% | 58.0 | 24-h Holter | Mean of 2 | ≥30% PACs/24h (Dic) | UV HR 2.60 (1.70-4.70)  MV HR N/A | **Total cohort:**  245/6,693 (3.7%)  **≥30% PACs/24h group:**  40/473 (8.5%)  **<30% PACs/24h group:**  205/6,220 (3.3%) |
| Cheriyath  2011^33^ | P | 14,574 | Mean of 54 | 43.0 | 2-min ECG | 14 (12.5-15.5) | ≥1 PAC (Dic) | UV HR 2.06 (1.39-3.07)  MV HR 1.48 (0.96-2.28) | **Total cohort:**  288/14,574 (2.0%)  **≥1 PAC group:**  27/716 (3.7%)  **No PACs group:**  261/13,685 (1.9%) |

DCAD – death from coronary artery disease; Dic – dichotomous; MV – multivariate (adjusted); N/A = not available; P – prospective; PAC(s) – premature atrial contraction(s); PYs – person-years; R – retrospective; UV – univariate (unadjusted)

† Values obtained by Huang *et al.*
